# 

**Published:** 1889-10

**Authors:** 


### TABLE OF CONTENTS.

ORIGINAL AND SELECTED.
Radical cure of fistula in ano and Hemorrhoids by electricity, by W S. Shotwell, M. D. ........ 361
Coma, by J. F Williams, M. D ..... 362
Scarlet fever; Successful case of intubation 364
Hydrophobia .... .... ..... ........ 366
On our knowledge of thiol ... ... .......... 368
Local Anæsthesia ... ........ ..... ... 370
Study of Brown-Sequard theory... .......... 372
ABSTRACTS &c.
Chloroform as a routine anæsthetic; Cocaine Poisoning .. .... ......... 377
Salicylates in rheumatism; Intubation .... 378
Iodized glycerin; Adherent prepuce; Drunkards in Norway; Novel treatment of Tuberculosis ... .... 379
Anemone Pulsatilla in the treatment of uterine affections... ... ... ..... ..... 380
Opium poisoning in an infant ..... .......... 381
Treatment of eczema in children ...... 382
Herpes circinatus; What semen has done for Brown-Sequard; Antipyrin epilepsy 383
Cure of nocturnal incontinence; Note on some gynecic uses of boric acid ......... .... 384
Dr. Beek writes; Leprosy and syphilis .. 385
New method of illuminating the larynx; Testicles as reinvigorators .... ... ............ 386
Treatment of ganglions; Brown-Sequard; Treatment of ortorrhœa ....... ..... . 387
Sunflower in malarial fevers; Treatment of carbuncles ........... ........ 388
Grafts of frog skin in chronic ulcers; More experience with the elixir of life............. . 389
SCIENTIFIC.
The conflict; Experiments; Automatic reading lamp .. .......... 390
The mosquito; Sixty years ago; Magnesium... 391
NOTES AND FORMULÆ.
Vinum compositum; Treatment of headache; Swift's Specific; To remove freckles; Antiseptic cotton ....... ........ 392
Exalgine; Osmic acid for sciatica; Cod-liver oil; A writer ..... ........ 393
EDITORIAL.
Dr. Burroughs and his borrowed discovery..... 394
Ovariotomy ..... ...... 395
Duodeno-cholecystostomy; Book notices ..... 396
Book notices; American Public Health Association; So Surg. and Gyn. Asso. ....... 397
American Rhinological Association in Europe; To our readers; Rectal troubles; Tri-State Medical Association call....... ......... 398
Special notes ......... ..... ...... 399, 400

